# Social and physical environmental correlates of independent mobility in children: a systematic review taking sex/gender differences into account

**DOI:** 10.1186/s12942-018-0145-9

**Published:** 2018-07-03

**Authors:** Isabel Marzi, Yolanda Demetriou, Anne Kerstin Reimers

**Affiliations:** 1 Faculty of Behavioral and Social Sciences, Chemnitz University of Technology, Chemnitz, Germany; 2 Department of Sport and Health Sciences, Technical University of Munich, Munich, Germany

**Keywords:** Independent mobility, Social environment, Physical environment, Children, Sex/gender differences

## Abstract

**Background:**

Children’s independent mobility (CIM) is an important contributor to physical activity and health in children. However, in the last 20 years CIM has significantly decreased. To develop effective intervention programs to promote CIM, the impact of the environment on CIM must be identified. This review seeks to provide an overview of sex/gender-specific socio-ecological correlates of CIM.

**Methods:**

A systematic literature search of five databases (PubMed, PsycInfo, Scopus, Medline, Web of Science) was conducted with a priori defined eligibility criteria and identified 1838 potential articles published between January 1990 and November 2017. Two independent reviewers screened the literature and identified and rated methodological quality of the studies. Related factors of CIM were summarized separately for CIM license (parental permission to travel independently) and CIM destination (destinations to which a child travels independently), and separately for boys and girls using a semi-quantitative method.

**Results:**

Twenty-seven peer-reviewed journal articles were identified which examined the relationship between the social and physical environment and CIM. Only seven studies reported results divided by sex/gender. Most associations between the environment and CIM were found in the expected direction (positive or negative) or not associated at all. The social environment seemed to be more influential for ensuring CIM than the physical environment. Neighborhood safety, fear of crime and stranger, parental support, and perception of traffic were important social environmental factors influencing CIM, while car ownership, distance, and neighborhood design were relevant physical environmental attributes. Few studies examined sex/gender-related environmental correlates of independent mobility, and those findings were inconsistent.

**Conclusion:**

The findings of this systematic review serve as suggestions for intervention programs to increase CIM and to identify future directions in research. To establish a robust comprehension of the impact of the social and physical environment on CIM, further sex/gender-sensitive studies using comparable measurements for CIM and environmental correlates are needed.

**Electronic supplementary material:**

The online version of this article (10.1186/s12942-018-0145-9) contains supplementary material, which is available to authorized users.

## Background

Physical activity is associated with numerous health benefits [1, 2]. However, in 2010 more than 80% of school-aged children worldwide did not to meet the World Health Organization recommendation of 60 min of moderate to vigorous-intensity physical activity daily [3, 4]. Walking or cycling for transport, otherwise known as ‘active travel’, is one way in which children can increase their levels of physical activity. A number of studies have examined the contribution of active travel to overall activity levels and health [5] and have generally found that children who walk to school are more likely to engage in physical activity overall and are more likely to meet physical activity guidelines than children who travel motorized. Additionally, children who walk or cycle to school have a lower BMI than those who are passive travelers [6].

Children’s independent mobility (CIM) defined as “the freedom of children to travel around their neighborhood or city without adult supervision” [7] is one important contributor to active travel and underscores the relationship between active travel behavior and physical activity. For both boys and girls, CIM is positively associated with physical activity on weekdays and, furthermore, for girls on weekends [8].

In addition to these health outcomes, CIM is associated with cognitive and motor development as well as social competencies of children [9–12]. Rissotto and Tonucci [13] showed that CIM has positive effects on cognitive development of children due to social and environmental experience. Furthermore, children who are independently mobile have more social competencies as they spend more time with peers than others [12]. In contrast, a lower CIM level predicts greater feelings of loneliness [14].

However, CIM has significantly decreased over the past 20 years [15, 16]. In Australia, the proportion of children travelling to school independently was 61% in 1991 but this proportion declined to 32% by 2012 [15]. Increased car use corresponding to a decline in independent mobility was recorded in several countries [17]. In Denmark, car use doubled between 1978 and 2000; within the same period the number of children walking to school fell by almost 40% [17]. Although Finnish children still enjoy the highest amount of independent mobility [18], CIM significantly decreased over a period of 20 years in Finland as well [16]: In the inner city of Helsinki, the proportion of children travelling independently to and from school decreased from 82 to 50%.

Socio-ecological models postulate multiple environmental influences on health behavior [19]. Children in particular are less autonomous concerning their physical activity and mobility and are more likely to be influenced by their environment than are adults [20]. Thus, understanding social and physical environmental correlates of independent mobility among children is an important prerequisite to develop effective interventions to increase the number of children engaging in independent mobility. Empirical studies examined various physical environmental (e.g., walkability, and urbanity) and social environmental (e.g., parental fear, perception of danger, and social support) factors that influence CIM. Changes in children’s physical environments over the past 20 years, such as more car traffic and fewer playgrounds, affect active travel behaviors and deter CIM [17, 21, 22]. Access to organized leisure activities determines the extent of CIM as Fyhri, Hjorthol [17] reported that children are often taken to leisure activities by car, because activities take place outside the immediate neighborhood. Additionally, due to an increasing crime rate, high urbanization, and long distances to school, parents limit CIM by prohibition [17, 23]. Moreover, the neighborhood environment and the local social network determine CIM [24]. Mothers’ perception of social danger and traffic around school has also been found to inhibit independent active travel [25, 26]. In contrast, older siblings or dog ownership are associated with greater CIM as older siblings and dogs provide parents an increased sense of safety [27].

Accounting for sex/gender differences with regard to independent mobility is important. Gender theories postulate that such differences are due to socially determined gender roles; gender-typed patterns of behavior occur based on socialization processes [28, 29]. Generally, girls tend to be less physically active than boys and less inclined to participate in organized sports [3, 30, 31]. Regarding CIM, sex/gender differences appear to exist, with girls having less freedom to travel around without parental supervision than boys [24, 32, 33]. Furthermore, boys seem to become independently mobile earlier than girls: Brown et al. [34] showed that 60% of boys between 4 and 6 years living in England are allowed to go out alone whereas the proportion of girls stands around 44%. As CIM is related to physical activity, knowing the reasons for low levels of CIM and considering them in intervention programs could be one way to increase physical activity in girls. There is evidence for different mechanisms explaining sex/gender differences in CIM: The higher protectiveness of parents about their daughters than about their sons and higher safety concerns can limit girls’ independent mobility level [25, 34–36].

Health promotion programs are increasingly designed based on the socio-ecological perspective, thus identifying various levels of contextual influences on children’s independent mobility is required [37, 38]. Nevertheless, to the best of our knowledge no comprehensive overview of socio-ecological correlates of independent mobility in children has yet been published. For active—but not independent—travel a review published by Panter et al. [39] pointed out the importance of environmental determinants of active travel behaviors. However, for CIM no such summary is available as a recent meta-analytical review by Sharmin and Kamruzzaman [40] focused solely on the association between the built environment and CIM. A systematic review by Qui and Zhu [41] focused on housing and community environments and its impact on CIM. Nevertheless, this review has its limitations, because no distinction was made between different types of CIM, i.e., range, destination, time or license and no quality assessment was conducted to identify current research gaps. Additionally, sex/gender-related differences concerning the correlates of CIM have not been incorporated in previous reviews [40, 41]. Thus, this systematic review aims to provide an overview of socio-ecological correlates of CIM with a particular focus on differences between boys and girls and categorized by different CIM types.

## Methods

This systematic review was conducted and is reported based on the Preferred Reporting Items for Systematic Reviews and Meta-Analyses (PRISMA) guidelines [42].

### Search strategy

The literature search was conducted on 7 November 2017 using the databases PubMed, Medline, Scopus, PsycInfo and Web of Science Core Collection. The search strategy included a combination of terms for independent mobility (“independent mobil*”), correlates (environment* OR neighborhood OR family OR families OR home OR parent* OR mother* OR father* OR sibling* OR urban* OR park*) and children (kids* OR child* OR girl* OR boy*). Additional articles were sought by reviewing reference lists of included full text articles and citations of full text articles using the Web of Science “Citation Network” statistics of each study.

### Eligibility criteria

Studies were deemed eligible if they met all following inclusion criteria: (1) subjects of the study were healthy children (age 3–12 years or the average age was in this range); (2) at least one association between CIM and an environmental (social and physical) correlate was examined; (3) an appropriate study design was used (cross-sectional or longitudinal; no case or intervention study); (4) the study employed a quantitative design; (5) the study was published in a peer-reviewed journal, written in English or German language; (6) the study was published after 1990, because in that year Hillman et al. [22] introduced the term “children’s independent mobility”, in their seminal study on this topic. An exception was made for some intervention studies if a cross-sectional analysis of the association of interest was reported.

If the study examined attitudes towards independent mobility instead of CIM itself, it was excluded. Studies referring to active commuting to school or active travel were only included if they clearly defined whether children travelled independently.

### Environmental correlates

Social and physical correlates were selected based on the socio-ecological model of Sallis et al. [19]. Correlates of the social environment were categorized into three subcategories: children’s perceived neighborhood environment (e.g., fear of stranger), parents’ perceived neighborhood environment (e.g., neighborhood friendliness), and social cultural environment (e.g., parental rules towards CIM). To categorize correlates of the physical environment the following five domains were established based on Sallis et al. [19] and Ding et al. [43]: home environment (e.g., car ownership), school environment (e.g., school-specific walkability), recreational environment (e.g., access to parks and playground), neighborhood design (e.g., degree of urbanization), and transport environment (e.g., traffic). Studies with only socio-demographic characteristics of the child, the family and/or household were excluded.

### Study selection

The study selection occurred in three steps compromising (1) title-screening, (2) abstract-screening, and (3) full-text-screening by two independent researchers (IM, CS). Studies were included or excluded depending on the eligibility criteria. During each step of the screening process, all references that could not be conclusively excluded were kept for further screening in the next step. Disagreement between the two reviewers on final inclusion was resolved by discussion with a third researcher (AKR). The selection process was documented using the reference management software EndNote X7 [44].

### Data extraction

The following data was extracted from each article: author(s); year of publication; country; study design; sample description (number of participants, age, sex/gender); definition, measurement, and instrument of CIM; type, measurement, and instruments of examined correlates; and main study results on the relationship between social and physical environmental factors and CIM (see Additional file 1). CIM was classified as CIM range, CIM time, CIM destination, or CIM license [40]. CIM range describes the distance children can travel independently from their home. CIM time defines how many minutes children can travel outside of their home independently. Destinations a child independently travels to are included in the term CIM destination. Whether parents allow children to travel independently is defined as CIM license.

### Quality assessment

The methodological quality of the studies included was evaluated by two independent reviewers (IM, KB) using 11 a priori defined quality criteria based on existing quality assessments published by Downes et al. [45] and Uijtdewilligen et al. [46]. Each criterion was either coded as “no” or “unclear” (0) if the study either did not meet the criterion or the criterion was not mentioned. If the study provided information on the quality item but only in parts, the criterion was coded as “partial” (0.5), while if the study completely met the criterion it was coded as “yes” (1). If a study referred to another publication containing relevant information for scoring the quality items, the study of interest was consulted. However, if the additional source did not provide the requested information or just in parts, the criterion was coded—according to the defined coding system—with “no” or “unclear”, respectively. As many studies included multiple correlates (e.g., social factors, and physical factors), criterion eight was scored on a scale ranging from 0 to 1. For example, if a study analyzed four different types of correlates of which three were measured with a reliable tool, a score of 0.75 was calculated. The methodological quality score of each study was calculated by the percentage of fulfilled criteria relative to the sum of all criteria (11 points in total). A quality score of ≥ 70% was considered high methodological quality, while a score of < 70% was considered insufficient methodological quality [46]. The quality assessment for each study is presented in Additional file 2.

### Synthesis of results

Due to the heterogeneity of social and physical environmental correlates and outcome measures of CIM a meta-analysis of the selected studies was considered inappropriate. The results of all selected studies were analyzed using a semi-quantitative method. In addition to associations between the social and physical environment and independent mobility in children in general, associations were considered separately for both, girls and boys, with appropriate studies. Bivariate associations between CIM and environmental correlates (19 studies) and multivariate regression models (20 studies) were considered separately as various socio-demographic, social, and physical environmental correlates were integrated into multiple regression models. As no study evaluated CIM time and only three CIM range, the results were only analyzed separately for CIM destination and CIM license.

The strength of evidence was adapted from previously published scoring systems [11, 43, 47]. If 0–33% of studies showed a significant association (p ≤ 0.05) of CIM and social or physical environmental correlates, the findings were classified as no association (0). If 34–59% of studies demonstrated significant associations, the findings were classed as being inconsistent (?). If 60–100% reported significant associations between CIM and the social or physical environment, the findings were categorized as positive (+) or negative (−), depending on the direction of the relationship. For less than four available studies for positive or negative associations the evidence was rated as limited (small +, −). Additionally, the methodological quality of the studies was included in scoring the strength of evidence. If 60–100% of high quality studies showed a significant correlation, the findings were considered strong evidence for a positive (++) or negative (−) correlation.

As the publications of Foster et al. [36], Villanueva et al. [48] and Villanueva et al. [33] analyzed the same study population, some social and physical environmental correlates were doubled in the results. For that reason significant associations of doubled correlates of these publications were considered as one study result for scoring the strength of evidence.

## Results

### Flow chart

**Fig. 1:**
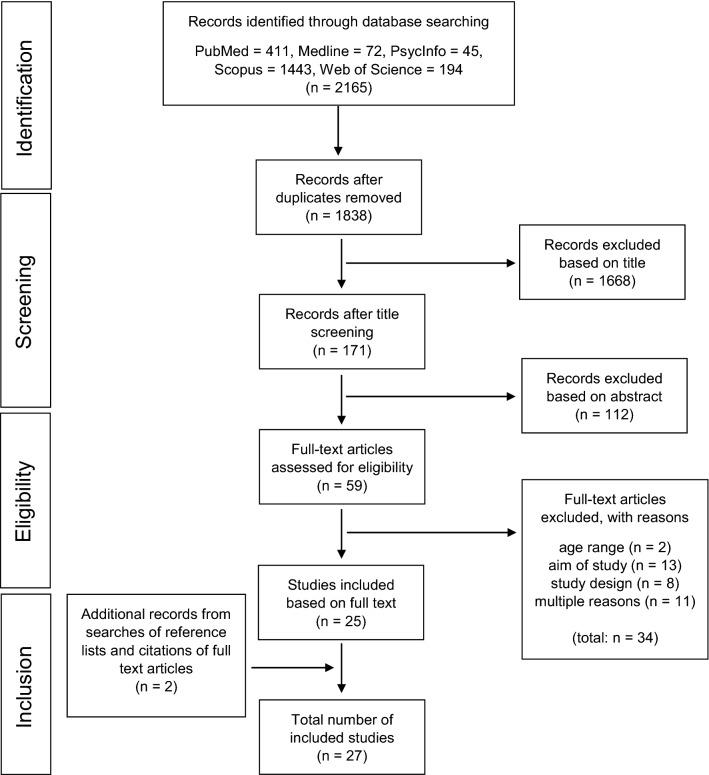
Flow chart

A total of 1838 potentially relevant articles (2165 including duplicates) were identified by the database search and screened based on title and abstract. Next, the full texts of 59 studies were retrieved for detailed review. As 34 studies were excluded due to inappropriate age range, aim of study, study design or for multiple reasons, 25 studies identified by the database screenings have been included in this systematic review. Two additional relevant publication were identified by backward reference tracking, yielding a total of 27 papers included in this systematic review (Fig. 1).

### Characteristics of included studies

General study characteristics are summarized in Table 1. An additional file shows more details of the studies included (see Additional file 1). More than 80% of the selected studies were cross-sectional, two were longitudinal and another two studies were longitudinal including cross-section analyses. The sample sizes ranged from 181 children [49] to a study population of 2110 children [50]. Nearly half of the studies were conducted in Europe, with 10 from either Australia or New Zealand, five from North America or Canada and one from Asia. Most studies (85%) were published between 2010 and 2017 with the earliest publication in 2001 [12].

**Table 1:** Characteristics of studies included (n = 27 studies)

Characteristics	N (%)	Study source
Study design		
Cross-sectional	23 (85)	[12, 23–26, 32, 33, 35, 36, 48, 50, 51, 53–59, 61–64]
Longitudinal including cross-sectional analyses	2 (8)	[27, 49]
Longitudinal	2 (8)	[52, 60]
Sample size		
< 500	14 (52)	[12, 25, 27, 49, 53, 55–61, 63, 64]
> 500	13 (48)	[23, 24, 26, 32, 33, 35, 36, 48, 50–52, 54, 62]
Geographic origin		
Europe	11 (41)	[12, 23, 25, 35, 51, 52, 56, 58, 59, 62, 64]
North America/Canada	5 (18)	[24, 26, 32, 61, 63]
Australia/New Zealand	10 (37)	[27, 33, 36, 48, 49, 53–55, 57, 60]
Asia	1 (4)	[50]
Publication year		
2010–2017	23 (85)	[23–27, 32, 33, 35, 36, 48–55, 57–61, 63]
1990–2009	4 (15)	[12, 56, 62, 64]
IM definition^a^		
CIM range	3 (11)	[35, 56, 63]
CIM time	0	
CIM destination	18 (67)	[23, 25, 26, 32, 33, 36, 48, 51–55, 57–60, 62, 64]
CIM license	10 (37)	[12, 23, 24, 27, 49, 53, 56, 58, 61, 64]
No response	1 (4)	[50]
IM Measurement		
Child reported	6 (22)	[51, 52, 55, 59, 60, 62]
Parent reported	10 (37)	[12, 24–27, 35, 49, 56, 61, 63]
Child and parent reported	10 (37)	[23, 32, 33, 36, 48, 53, 54, 57, 58, 64]
No response	1 (4)	[50]
Correlates measurement		
Objective	1 (4)	[51]
Subjective	10 (37)	[12, 24–27, 35, 56, 59, 62, 63]
Objective and subjective	16 (59)	[23, 32, 33, 36, 48–50, 52–55, 57, 58, 60, 61, 64]

^a^More than 100% possible due to multiple types of CIM in one study

In the majority of studies, the mean age of the sample population ranged from 9 to 12 years [12, 23–25, 27, 32, 33, 35, 36, 48, 49, 51–61]. Fewer studies focused on younger children aged 6 to 9 years [26, 50, 59, 62–64]. All studies targeted girls and boys, but merely seven studies separated results by sex/gender [33, 35, 36, 48, 52–54]. Of all studies, eighteen described CIM by destinations a child traveled independently to. Fewer studies examined CIM licenses. CIM range was evaluated by another three studies. No study evaluated CIM as independent time outside. Ten studies utilized solely parent-report measures of CIM, and a further six studies applied children’s self-reporting. Another ten studies combined child and parental report measurements of CIM. Twenty-one studies [12, 23–25, 32, 33, 35, 36, 48, 49, 52–57, 60–64] examined both social and physical environmental correlates of CIM. Three studies [50, 51, 58] focused on the relationship between the physical environment and CIM and two further studies [26, 59] reported only the association between social environmental factors and CIM. For social and physical environmental factors most studies combined objective and subjective measurements. Ten studies applied only subjective measurements and one study used solely objective measurements.

### Results of methodological quality assessment

The study quality was rated high in 12 studies [25, 27, 32, 36, 49, 51, 52, 55, 59–61, 64] and low in 15 studies [12, 23, 24, 26, 33, 35, 48, 50, 53, 54, 56–58, 62, 63]. The agreement between the two reviewers in the methodological quality of included studies (IM and KB) was 75% (Cohen’s kappa κ = 0.752). In discussion of individual study quality scores, a final agreement of 100% was achieved. The methodological quality criteria and the number and proportion of studies fulfilling the criteria are presented in Table 2; more detailed quality assessments of each study included is presented in an additional file (see Additional file 2). All 27 studies contained clearly defined aims. As the aim of the majority of the studies was to identify causal relationships between the social and physical environment and CIM, most studies did not fulfil the criterion regarding study design. Twenty-five studies failed to meet criteria for response rate, since the response rate was less than 80% or not clearly defined. Only three studies undertook measures to address and categorize non-responders. Standardized methods of acceptable quality were used to measure CIM and correlates in more than 50% of the studies. One study lacked clearly defined statistical methods and internally consistent results. More than 80% of the studies presented results for all the analyses described in the methods; four studies were missing some data.

**Table 2:** Criteria for methodological quality assessment and number (%) of studies scoring points for each criterion

		Studies fulfilling the criteria n (%)	
		Yes	Partial
Criteria			
1	Were the aims/objectives of the study clear?	27 (100)	0 (0)
Methods			
2	Was the study design appropriate for the stated aims?	6 (22)	21 (78)
3	Were the main features of the study population stated (description of sampling frame, distribution by age and sex/gender)?	14 (52)	13 (48)
4	Was the response rate at least 80%?	2 (8)	0 (0)
5	Were measures undertaken to address and categorize non-responders?	3 (11)	0 (0)
6	Were the exposure and outcome variables measured appropriate to the aims of the study?	17 (63)	10 (37)
7	Were standardized methods of acceptable quality used to measure IM?^a^	12 (44)	6 (22)
8	Were standardized methods of acceptable quality used to measure correlates?^a^	18 (69)	6 (22)
9	It is clear what was used to determine statistical significance and/or precision estimated (e.g., p values, confidence intervals)?	23 (85)	2 (8)
Results			
10	Were the results internally consistent?	26 (96)	1 (4)
11	Were the results presented for all the analyses described in the methods?	23 (85)	4 (15)

^a^Reliability: ICC > 0.70; Cronbach’s alpha > 0.65, pilot testing, published previously

### Social environment and CIM destination

A total of 48 associations of social environmental correlates and independent mobility to different destinations were reviewed in 10 studies. Some studies investigated similar constructs but employed different terminologies. Thus, these variables (e.g., safe for children, and safe place) were collected and an umbrella term was used (e.g., neighborhood safety) yielding a total sum of 23 social environmental correlates (Table 3). Three of four correlates describing children’s perceived neighborhood environment were significantly associated with CIM destination in the expected direction: fear of strangers (67%), neighborhood safety (100%), and many other children residing within their area (100%). Concerning the parental perceived neighborhood environment, 75% of all comparisons showed significant associations with CIM destination, including fear of strangers (100%), fear of crime (100%), neighborhood friendliness (100%), perception of traffic (63%), informal social control (100%), and people out on walks in the neighborhood (100%). Less evidence existed for associations between the social cultural environment and CIM destination; four of eight variables had more than 60% of significant associations. Mobility licenses (100%), confidence in children’s abilities (100%), a child’s personal safety (100%), and having friends (100%) were positively associated with CIM destination. Several relationships of social environmental correlates and CIM destination were categorized as inconsistent, including neighborhood friendliness (children; 50%), neighborhood safety (parents; 40%), parental rules (50%), and parent encouragement (50%). No association showed strong evidence with 60–100% of high quality studies reporting the association in the expected direction.

**Table 3:** Social and physical environmental correlates of CIM Destination

Correlates	Study source	Association with CIM			Strength of evidence	
		+	0	–	Association^a^	n/N (%)^b^
Social environment						
Perceived neighborhood environment (children)						
Fear of strangers	[32, 33]		[33] F	[33] M; [32]	–	2/3 (67)
Neighborhood friendliness	[33]	[33] M	[33] F		?	1/2 (50)
Neighborhood safety	[32, 33]; [62]^c^	[33] M, F; [32]		[62]^c^	+	4/4 (100)
Many other children within their area	[33, 36]	{[33] M, F; [36] M, F}^e^			+	2/2 (100)
Perceived neighborhood environment (parents)						
Sense of community	[52]		[52] (M, F)		0	0/2 (0)
Fear of strangers	[32, 36]			[32]; [36] M, F	–	3/3 (100)
Fear of crime	[63]			[63]	–	1/1 (100)
Neighborhood friendliness	[33, 36, 60]	{[33] M, F; [36] M, F}^e^; [60]			+	3/3 (100)
Neighborhood safety	[52, 63]; [52]^c^	[52] F; [63]	[52] M; [52]^c^ M, F		?	2/5 (40)
Perception of traffic	[36, 52, 62, 63]; [32, 33]^d(2)^; [33, 60, 63]^c^	[33]^c^ M, F; [63]^c^	[33] M, F; [52]M, F; [36] M; [60]^c^	[62, 63]; [32]^d(2)^; [36] F; [33] M, F	–	10/16 (63)
Often people out on walks in the neighborhood	[33]	[33] M, F			+	2/2 (100)
Informal social control	[36]	[36] M, F			+	2/2 (100)
Social cultural environment						
Mobility license	[53–55, 64]	[53] M, F; [54] M, F; [55, 64]			+	6/6 (100)
Parental rules (towards IM) walking	[52]		[52] M, F		0	0/2 (0)
Parental rules (towards IM) play outside	[52]	[52] M	[52] F		?	1/2 (50)
Parent encourage for walking/cycling	[52]		[52] M	[52] F	?	1/2 (50)
Friend encourage for walking/cycling	[52]		[52] M, F		0	0/2 (0)
Confidence in children’s abilities	[33, 60]	[33] M, F; [60]			+	3/3 (100)
Child’s personal safety	[33, 60]	[33] M, F; [60]			+	3/3 (100)
Fearful of child engaging in antisocial behavior	[33]		[33] M, F		0	0/2 (0)
Parental physical activity	[63]		[63]		0	0/1 (0)
Parent activity with child	[63]		[63]		0	0/1 (0)
Many children we know walk or cycle to school	[60]		[60]		0	0/1 (0)
Having friends	[33]	[33] M, F			+	2/2 (100)
Physical environment						
Home environment						
Car ownership	[32, 50, 52, 62]		[32]; [52] F	[50, 62]; [52] M	–	3/5 (60)
Dog ownership	[27]	[27]			+	1/1 (100)
Bike ownership	[33]	[33] F	[33] M		?	1/2 (50)
Size of backyard	[33]		[33] M, F		0	0/2 (0)
School environment						
Distance	[32, 50, 51, 62]			[32, 50, 51, 62]	–	4/4 (100)
School-specific walkability	[33, 36, 52, 62]	{[33] F; [36] F}^e^; [62]	{[33] M; [36] M}^e^; [52] M, F		?	2/5 (40)
School characteristics	[52]		[52] M, F		0	0/2 (0)
School density	[50]	[50]			+	1/1 (100)
Recreational environment						
Parks	[33, 60]	[33] M	[33] F; [60]		?	1/3 (33)
Quality and quantity of public open spaces	[55]		[55]		0	0/1 (0)
Remote places	[51]	[51]			+	1/1 (100)
Neighborhood design						
Street connectivity	[32, 50]; [52]^d (3)^		[52] M, F; [52] (M); [52] M, F	[32, 50]; [52] F	?	3/8 (38)
Neighborhood walkability	[52]		[52] M, F		0	0/2 (0)
Land use mix	[32, 50, 52]		[52] M; [50]	[32]; [52] F	?	2/4 (50)
Population density	[50]			[50]	–	1/1 (100)
Degree of urbanization (ref: urban)	[23, 50, 53, 54, 58, 62, 64]	[54]	[53]	[23, 50, 58, 62, 64]	–	5/7 (71)
Urban structure (new)	[32]	[32]			+	1/1 (100)
Street-trees	[32]		[32]		0	0/1 (0)
Densely built up residential areas	[51]	[51]			+	1/1 (100)
Mainly single-family housing	[51]	[51]			+	1/1 (100)
Big building and public transport hubs	[51]			[51]	–	1/1 (100)
Transport environment						
Walking facilities	[52]; [60]; [32]^c^	[60]	[52] M, F	[32]^c^	?	2/4 (50)
Biking facilities	[60]		[60]		0	0/1 (0)
Streetlight density	[52]		[52] M, F		0	0/2 (0)
Traffic (objective)	[50, 51]; [32]^d (2)^		[32, 51]	[32, 50]	?	2/4 (50)

Effects which are specific to different sex/gender groups are noted separately: M (male); F (female)

*CIM* children’s independent mobility

^a^No evidence: no studies were identified; no association (0): 0–33% of studies showed a significant association; inconsistent association (?): 34–59% of studies reported significant associations; positive (+) or negative (−) association: 60–100% of studies demonstrated significant associations; limited evidence for a positive or negative association (small +, −): <4 studies available for the associations of interest; strong evidence (++) or (−−) association: 60–100% of high quality studies showed a significant association

^b^n = number of studies/measures reporting associations in the expected direction; N = number of identified studies/measures on the association of interest; (%) = percentage of studies reporting associations in the expected direction

^c^Items are reversed

^d(x)^The same study may occur twice or more often within a topic if different measures are used and show different associations; x = number of measures

^e^{…} = study results of two studies with the same population were considered as one study

### Physical environment and CIM destination

The evidence for associations between the physical environment and CIM destination was much weaker than for social environmental correlates. Only 11 of 23 variables demonstrated evidence for significant associations with CIM destination, reviewed in 14 studies (Table 3). Car ownership (60%), dog ownership (100%), distance to school (100%), school density (100%), remote places (100%), population density (100%), degree of urbanization (71%), urban structure (100%) were consistently associated with CIM destination in the expected direction, but with little evidence. Furthermore, different neighborhood designs, such as mainly single-family housing, densely built up residential areas and big buildings were associated with CIM. Inconsistent associations were reported for five physical environmental variables, including bike ownership (50%), school-specific walkability (40%), access to parks (33%), street connectivity (38%), and land use mix (50%). Transport attributes, including walking facilities, biking facilities, streetlight density and traffic (objective), were also not consistently associated with CIM destination.

### Social environment and CIM license

With regard to children’s license to be independently mobile, fewer correlates were examined due to a limited number of studies (7 studies). In total eight correlates regarding the parental perceived neighborhood environment and social cultural environment were reviewed (Table 4). Perceived neighborhood attributes, such as fear of strangers (100%), neighborhood friendliness (66%), and neighborhood safety (100%), were significantly associated with CIM license. Additionally, social norms (100%) and parents’ travel attitudes (100%) showed associations with CIM license. All associations demonstrated little evidence. The associations between CIM license and parents perceived fear of crime (34%) and traffic (34%) were inconsistent.

**Table 4:** Social and physical environmental correlates of CIM License

Correlates	Study source	Association with CIM			Strenght of evidence	
		+	0	–	Association^a^	n/N (%)^b^
Social environment						
Perceived neighborhood environment (parents)						
Fear of strangers	[24]^c^	[24]^c^			+	1/1 (100)
Fear of crime	[61]^c;^ [49]^d (2)^	[61]^c^	[49]^d (2)^		?	1/3 (33)
Neighborhood friendliness	[24, 61]; [49]^c^	[24, 61]	[49]^c^		+	2/3 (66)
Neighborhood safety	[24, 61]; [49]^c^	[24, 61]		[49]^c^	+	3/3 (100)
Perception of traffic	[24, 49]; [61]^c^	[61]^c^	[24, 49]		?	1/3 (33)
Neighborhood maintenance	[49]^d(4)^		[49]^d(4)^		0	0/4 (0)
Social cultural environment						
Social norms (no support of IM)	[49]			[49]	–	1/1 (100)
Parents’ attitudes toward active travel modes	[24]	[24]			+	1/1 (100)
Child-centered social control	[61]		[61]		0	0/1 (0)
Physical environment						
Home environment						
Car ownership	[61]			[61]	–	1/1 (100)
Recreational environment						
Park availability	[49]^d (2)^		[49]^d (2)^		0	0/2 (0)
Park attractiveness	[49]	[49]			+	1/1 (100)
Playgrounds	[49]^d (2)^		[49]^d (2)^		0	0/2 (0)
School environment						
School density	[49]^d (2)^		[49]^d (2)^		0	0/2 (0)
Neighborhood design						
Housing unit density	[61]		[61]		0	0/1 (0)
Degree of Urbanization	[23, 53, 58, 64]		[53]	[23, 58, 64]	–	3/4 (75)
Neighborhood Walkability	[24]^d(6)^		[24]^d(6)^		0	0/6 (0)
Transport environment						
Traffic (objective)	[49]		[49]		0	0/1 (0)

*CIM* children’s independent mobility

^a^No evidence: no studies were identified; no association (0): 0–33% of studies showed a significant association; inconsistent association (?): 34–59% of studies reported significant associations; positive (+) or negative (−) association: 60–100% of studies demonstrated significant associations; limited evidence for a positive or negative association (small +, −): <4 studies available for the associations of interest; strong evidence (++) or (−−) association: 60–100% of high quality studies showed a significant association

^b^n = number of studies/measures reporting associations in the expected direction; N = number of identified studies/measures on the association of interest; (%) = percentage of studies reporting associations in the expected direction

^c^Items are reversed

^d(x)^The same study may occur twice or more often within a topic if different measures are used and show different associations; x = number of measures

### Physical environment and CIM license

Similar to CIM destination less significant associations existed for physical environmental attributes and CIM license; only three of nine variables demonstrated significant associations. As shown in Table 4, car ownership (100%), park attractiveness (100%), and degree of urbanization (75%) were associated with CIM license in the expected direction. Strong evidence was not found for any association with physical environment. No variable of the school environment and transport environment was associated with CIM licenses and neither was availability of parks and playgrounds.

### Results from multivariate regression models

The results of the multivariate regression models of 12 studies, including these models in addition to univariate associations, and of eight studies solely presenting multivariate regression models were also analyzed in this systematic review (Table 5). These results highlight the evidence of bivariate associations between the environment and CIM. Nevertheless, socio-demographic characteristics such as age and sex/gender remained significant in nearly all regression models and tended to be important predictors of CIM. Additionally, having siblings was significantly associated with CIM in four models. Concerning the social environment, perceived neighborhood attributes, such as neighborhood safety and fear of strangers, remained significant correlates of CIM. Moreover, parental rules and attitudes towards independent mobility showed associations with CIM. Three physical environmental attributes were consistently associated with CIM: distance to school, car ownership and traffic. There is less evidence for neighborhood design (e.g., walkability) and degree of urbanization, but many models were controlled for urban setting and, thus, did not report this association.

**Table 5:** Results of multivariate regression models showing only significant correlates of CIM (n = 20 studies)

Study source	Socio-demographic and psychosocial characteristics	Social environment	Physical environment
Alparone et al. [25]	Age, birth order	fear of strangers Perception of positive potentiality of outdoor autonomy	n. s.
Buliung et al. [32] (to school)	Age,sex/gender Flexible work schedule	Neighborhood safety	Distance Traffic
Buliung et al. [32] (from school)	Age, sex/gender, Flexible work schedule, fathers’ employment status	Neighborhood safety	Traffic
Christian et al. [49]	Age, older siblings	Neighborhood safety Social norms	n. s.
Cordovil et al. [23]	Age	Mobility license	Car ownership Distance
Fyhri et al. [62]	Age, sex/gender	Neighborhood safety Fear of strangers	Parents car use frequency Distance
Janssen et al. [63]	Age	Neighborhood safety Fear of crime	n. s.
Johannson [56] CIM license	Age, maturity, siblings	Neighborhood safety Traffic perception Need to protect	n. s.
Johannson [56] CIM range	Age, maturity, siblings	Attitude towards CIM	Car ownership Traffic
Kytta [16] (Finnish data)	n. s.	Mobility license	Urbanization
Kytta [64] (Belrushian data)	Sex/gender	n. s.	Urbanization
Lam and Loo [50]	Age Household income, family structure, mothers’ employment status, domestic helpers at home	n. s.	Distance Urbanization School density
Lin et al. [57]	Siblings	n.s.	Car ownership Distance
Mammen et al. [26]	Age Language spoken at home	Fear of strangers Traffic perception	Car ownership Distance
Prezza et al. [12]	Age, sex/gender	Neighborhood Friendliness	Park accessibility Urban structure Courtyard
Santos et al. [59]^a^	n. r.	Neighborhood safety Parents’ physical activity	
Veitch et al. [60]^b^ school	Child enjoys walking	child’s personal safety	Walking facilities
Veitch et al. [60]^b^ local destinations	n. s.	Many other children with in the neighborhood	n.s.
Wolfe and McDonald [61]	Age, race	Neighborhood safety	Housing unit density
Multivariate regressions models with separate results for boys and girls			
Carver et al. [52]^d^ girls	n. r.	Parent encouragement for walking	Street connectivity Land use mix
Carver et al. [52]^d^ boys	n. r.	Parental rules towards outdoor play	Car ownership
Foster et al. [36] girls	n. r.	Fear of strangers Informal social control	n. r.
Foster et al. [36] boys	n. r.	Fear of strangers	n. r.
Ghekiere et al. [35] girls	Grade Cycle skills Traffic skills	n. s.	traffic
Ghekiere et al. [35] boys	Grade Cycle skills Traffic skills	n. s.	n. s.
Villanueva et al. [35]^e^ girls	Child’s confidence	Neighborhood safety Traffic perception Confidence in child’s ability	Bike ownership School-specific walkability
Villanueva et al. [48]^e^ boys	Child’s confidence	Neighborhood friendliness Traffic perception confidence in child’s ability	Distance to green space Count of shopping centers, recreation venues, community services and retail shops Park attractiveness
Villlanueva et al. [48]^e^ girls	Child’s confidence	Neighborhood safety People on walks in the neighborhood Confidence in child’s ability	Bike ownership School-specific walkability
Villlanueva et al. [48]^e^ boys	Child’s confidence	Neighborhood safety Many other children with in the neighborhood Traffic perception confidence in child’s ability	

Abbreviations: *CIM* children’s independent mobility, *n. r.* not reported, *n. s.* not significant

^a^Adjusted for child’s age and gender

^b^Controlled for sex and age of child, urban/rural location, maternal education and employment, distance to school, whether the child changed school between T1 and T2, and clustering within suburbs

^c^Controlled for sex and age of child, urban/rural location, maternal education and employment, and clustering within suburbs

^d^Controlled for parental education level, distance from home to school, urban/rural

^e^Adjusted for socio-economic status, age, maternal education, child’s school year, whether or not child was sick last week, school clustering

### Sex/gender differences in CIM correlates

Due to a limited number of studies reporting results separately for boys and girls and the heterogeneity of correlates, no evidence can be found for sex/gender-specific correlates of independent mobility. Mobility licenses were positively associated with CIM for boys and girls. Two further correlates analyzed in two different studies [36, 52] were school-specific walkability and parental perception of traffic, which yielded inconsistent results for girls and boys (Table 3). Concerning the results of multivariate regression models, only for physical environmental attributes differences were reviewed between boys and girls. For girls only, neighborhood design, such as walkability and land use mix as well as access to a bike, was associated with girls’ independent mobility [48, 52]. Car ownership and destination accessibility (e.g., parks, shopping centers, and recreation venues)  were significant correlates of independent mobility in boys but not in girls [48, 52]. Inconsistent associations were reported for traffic with studies reporting associations for boys and girls and only for girls [33, 35, 48].

## Discussion

The aim of this systematic review was to identify social and physical environmental correlates of independent mobility in children with a special focus on sex/gender. Associations of CIM destination and license and social environment were consistently positive, except for fear of strangers and crime. Car ownership and urban setting showed consistently negative associations with CIM License and CIM destination, respectively. However, five physical environmental attributes (dog ownership, shorter distance to school, school density, remote places, and new urban structure) were positively associated with CIM destination. Differences in correlates of independent mobility in boys and girls were found solely for the physical environment, with neighborhood design and bike ownership influencing girls’ independent mobility and associations of destination accessibility and car ownership with boys’ independent mobility.

CIM is related to physical activity, e.g., if children are walking or cycling without adult accompaniment. However, correlates of CIM seem to be slightly different to correlates of physical activity [43]. CIM tends to be more determined by the social environment than the physical environment. In comparison, physical activity is more associated with objectively measured environmental attributes such as walkability and traffic speed/volume [43]. For CIM other factors may be important to address when developing intervention programs, such as social norms, parents’ perceptions of neighborhood, and parental rules. Particularly, CIM license depends on parents’ perception of neighborhood environment [24, 49, 61]. As many studies have demonstrated that CIM increases with children’s age [23, 25, 62], parental restrictions on CIM probably decrease with children’s age while physical environmental attributes which support independent active travel may gain in importance.

Previous reviews of the physical environment and children’s travel behavior reported inconsistent results and non-occurring associations for several physical attributes as well [39, 65]. For children, the impact of the physical environment is potentially influenced by granting (or not) of mobility licenses by their parents who are literally the gatekeepers of their travel behavior. For adults a variety of neighborhood physical features, such as walkability, street connectivity and access to services, are consistently associated with active travel behavior [66, 67].

The two constructs “parental perception of traffic” and “traffic (objective)” supported the conclusion that social environmental attributes determine CIM more than those of the physical environment: traffic perception was consistently associated with CIM; inconsistent associations were reported for objectively measured traffic. Nevertheless, physical environmental correlates with inconsistent results (e.g., school-specific walkability, access to parks, street connectivity, and land use mix) need to be addressed in future research as some studies have shown that activity-friendly environments can promote active travel behavior [68].

Results of multivariate regression models of studies included in this review underline the differing relevance of the social and physical environment for CIM. Age, sex/gender and parental perceived neighborhood environment tend to be represented as significant correlates of CIM [12, 25, 26, 32, 49, 50, 61, 62]. On the other hand, few physical environmental correlates (e.g., car ownership, and distance) remained significant in multivariate regression models [26, 32, 50, 52, 56, 57, 62]. Although these models help to identify the actual effects of different correlates on CIM, the comparison of findings should be interpreted carefully as all regression models integrated a wide range of socio-demographic, social and physical environmental correlates and adjusted for different variables.

Only three studies focused on parental physical activity and parents’ and friends’ encouragement for walking, but the results were inconsistent [52, 59, 63]. However, family and peer support and modeling seemed to be relevant for CIM as in other studies having siblings and friends was positively related to CIM [33, 49, 56, 57]. Previous reviews showed that parental physical activity and physical activity of peers is associated with youth activity behaviors [47, 69, 70] . Additionally, a study by Mackett et al. [71] demonstrated that girls in particular were only allowed to go out if they were accompanied by other children. In order to promote CIM, future research should specially focus on parent and peer related correlates of CIM, such as social support and social modelling. Furthermore, parent perceived environmental factors, such as fear of crime and neighborhood safety which were inconsistently associated with CIM, demand further research.

In physical activity, sport participation and CIM sex/gender differences are consistently reported, with girls tending to be less active or rather mobile than boys [3, 12, 72]. To overcome these sex/gender gaps, intervention programs need to be sex/gender-sensitive and take sex/gender-specific situations into account. A study by Reimers et al. [72] pointed out that girls residing longer destinations from the nearest sport facilities are less likely to take part in club sport activities. This relationship has not been observed in boys in this study. Promoting independent mobility in girls could therefore increase both their physical activity on the way to sports facilities and other physical activities by enabling girls to get access to other physical activity facilities or locations. CIM could be a door opener to get access to physical activity facilities and locations, such as gyms, playgrounds, parks etc., where children can participate in various physical activities and are able to meet other children to actively play with. This could be true for girls and boys. Promoting CIM in girls and boys could contribute to physical activity as active living behavior. Since an active living behavior is often established in childhood and adolescence, it could also affect physical activity and health in adulthood [73, 74].

Thus, this systematic review was the first to investigate sex/gender-related correlates of CIM for the development of effective intervention programs. Sex/gender differences with regard to the extent of independent mobility are consistently reported as being lower for girls than for boys [12, 34, 58]. To promote CIM, especially in girls, it is necessary to sufficiently understand the impact of social and environmental influences on girls’ and boys’ independent travel behaviors. However, only seven studies were identified that reported results separately for boys and girls [33, 35, 36, 48, 52–54]. Due to heterogeneity in the correlates and differing statistical methods, the results of the studies did not provide evidence of sex/gender-related correlates of CIM. This marks a research gap which must be addressed in future studies to develop effective sex/gender-specific intervention programs for boys and girls.

In this systematic review all studies with a quantitative design were included, irrespective of whether they were cross-sectional analyses or longitudinal analyses. Thus, the objective was to identify correlates of CIM because the number of high quality longitudinal studies was very limited. However to identify causal relationships between the environment and CIM, more longitudinal studies are needed.

According to Sharmin and Kamruzzaman [40], in the present systematic review, CIM was categorized into four different types: CIM destination, CIM license, CIM time, and CIM range. However, the majority of studies examined CIM destination and CIM license, and the results were solely presented for these two definitions of CIM. A study by Bhosale et al. [75] showed that CIM license and destination access are significantly correlated, which explains the similarities in the social and physical environmental correlates of both CIM types.

Apart from similarities between CIM license and CIM destination, some studies [49, 61] reported that the correlates of independent mobility differ between visited destinations. For example distance to park [49] and social control [61] were correlated with specific destinations (i.e., a park and a friend’s house, respectively) but not with overall independent mobility. Due to a lack of comparability, in this systematic review, the results were only analyzed for overall independent mobility. More research is required to separate correlates by visited destination.

CIM range was analyzed in merely three studies and CIM time not at all. Time and range may be insufficient indicators for CIM or simply rarely used at this stage. Using GPS as an objective measure of CIM could provide a more comprehensive understanding of CIM time and CIM range as indicator of CIM [76].

Based on the results of the methodological quality assessment, the lack of a standardized definition and measurement of independent mobility and environmental correlates limited the comparison of the included studies. In fact, a wide variety of measurements were used to determine CIM. Although results were separated for CIM license and CIM destination, measurements differed for child and parental report, with no study employing objective measures. To compare studies Bates and Stone [76] recommended using a standardized methodological design and a combination of subjective and objective measurements of CIM in future research.

Additionally, a wide selection of social and physical environmental correlates was used in the studies to evaluate the relationship between the environment and CIM. Thus, some correlates appeared only in one study, which provided only limited evidence. Furthermore, terminological differences were observed between researchers. For example, “stranger danger” was paraphrased as “trust in strangers” [24], “worried about strangers” [32, 33], and “fear of strangers” [32], which based on the description and measurement evaluated the same construct or the contrary. Due to such differences the comparison of various studies might be limited. Thus, future research should aim to create and apply standardized terms. Moreover, the heterogeneity of applied measurements for the same social and physical construct in different studies and the lack of reliable tools could lead to inconsistent findings as well.

The lack of generalizability of study results is caused by insufficient response rates in almost all studies. Acceptable response rates should to be at least 80% [46]. The response rate of included studies ranged between 18% [53] and 100% [64] with merely two studies above the recommended 80% [25, 64].

To analyze the influence of the methodological quality of studies on strength of evidence and the associations evaluated, differences in the results between high and low quality studies were considered. However, no systematic differences were found for positive, negative and non-existing associations in high and low quality studies. Thus, the methodological quality has no potential influence on inconsistent associations.

### Strengths and limitations

A strength of this review is the systematic search of relevant primary studies employing several search engines and a comprehensive list of search terms. Furthermore, the reference lists of all studies included were reviewed for additional sources. Another strength is that two independent reviewers (IM, CS) systematically screened relevant articles in three steps. Additionally, to evaluate the risk of bias a quality assessment was developed based on existing criteria lists for cross-sectional studies focusing on the methodological quality of the studies included. All results (bivariate and multivariate associations) were included in this systematic review, but analyzed separately as the multivariate associations integrated different correlates and, thus, were not directly comparable with the bivariate associations. Nevertheless, including both statistical methods helps to provide a further understanding of the relationship between socio-demographic characteristics, the social environment, and the physical environment with CIM. Stratifications of results by CIM definition provided additional information on the relationship between the environment and CIM.

A first limitation of this systematic review is the lack of evidence for causal relationships between the environment and CIM, because only two studies contained a longitudinal study design. Secondly, associations were only considered by significance and direction, not by effect size. Thirdly, conclusions may be heavily influenced by the low methodological quality of the studies and by individual findings of single studies, because many conclusions were based only on the result of one study. Additionally, no association demonstrated strong evidence, i.e., associations with at least four studies of high quality reporting the significant association in the expected direction. The fourth limitation is that only English- and German-language articles were considered for this review. Finally, as few studies reported sex/gender-specific results, sex/gender-related correlates could not be analyzed in detail.

## Conclusion

This systematic review provides an overview of social and environmental correlates of independent mobility in children and highlights important research gaps.

Based on the socio-ecological perspective, this systematic review pointed out important social and physical environmental correlates which could be considered when developing intervention programs to halt the decline of CIM, especially in girls. Overall, the synthesis of existing studies revealed that neighborhood safety, fear of crime and strangers, parental support and perception of traffic are significant social correlates. Furthermore, car ownership, distance, and neighborhood design belong to physical environmental attributes, which influence CIM. To possibly address factors, such as neighborhood safety or fear of crime, which are limitedly modifiable and more determined by political decisions concerning the domestic security, intervention programs should focus on the interaction between social and physical environment [77]. Promoting children to walk or cycle to school together with other children instead of walking alone and promoting their competence to travel safely could be one way to deter parent’s concerns about safety in the neighborhood and to expand mobility licenses [78].

Additionally, this systematic review identified future directions of research and suggests that the influence of the environment on CIM has not yet been fully understood. One important aspect is the implementation of longitudinal studies focusing on children’s independent mobility to get insights into causal relationships of the social and physical environment and CIM. Additionally, to foster a robust understanding of the impact of the social and physical environment on CIM, more studies employing comparable measuring standards for CIM and environmental predictors are needed.

Furthermore, this systematic review showed that sex/gender-related correlates are limitedly evaluated in literature until now. Promoting independent mobility, especially in girls, could additionally promote their physical activity and thus, contributes to healthy development in children [11]. To consider social inequalities in sport participation between boys and girls and the sex/gender gap in CIM future research should evaluate sex/gender-specific correlates of CIM.

## Additional files

**Additional file 1.** Characteristics of studies included on association of CIM and the social and physical environment The table shows the extracted data separated for all studies included, consisting of author(s); year of publication; country; study design; sample description (number of participants, age, sex/gender); definition, measurement, and instrument of CIM; type, measurement, and instruments of examined correlates; and main study results on the relationship between social and physical environmental factors and CIM.

**Additional file 2.** Quality Assessment for all studies included. The table shows the methodological quality rating of each criteria and the total quality score for all included studies.

## Abbreviation

CIM: children’s independent mobility
