# Supplementary material for: Social and physical environmental correlates of independent mobility in children: a systematic review taking sex/gender differences into account
Source: Int J Health Geogr. 2018 Jul 3;17:24. doi: 10.1186/s12942-018-0145-9 (PMC6029402; doi:10.1186/s12942-018-0145-9)
Supplement: Supplementary file 1 — Additional file 1. Characteristics of studies included on association of CIM and the social and physical environment The table shows the extracted data separated for all studies included, consisting of author(s); year of publication; country; study design; sample description (number of participants, age, sex/gender); definition, measurement, and instrument of CIM; type, measurement, and instruments of examined correlates; and main study results on the relationship between social and physical environmental factors and CIM. [file 12942_2018_145_MOESM1_ESM.pdf]

Additional file 1. Characteristics of studies included on association of CIM and the social and physical environment

No	Author<br>Year<br>Country	Study Design<br>Duration<br>Sample<br>(sample size, mean age/range, gender, response rate)	Dependent Variable (IM)<br><br>Definition<br>Measurements<br>Instruments	Independent Variable (types of environmental correlates)<br><br>Measurement<br>Instruments	Results
**1**	Alparone et al., 2012<br><br>Italy	**Study design**<br>Cross-sectional<br>**Duration**<br>n.r.<br>**Sample**<br>n = 313<br>*9.3 yrs*/8-10 yrs<br>51.4% F<br>RR: 81%	**Definition**<br>CIM destination<br>**Measurements**<br>Parental reports (mother)<br>**Instruments**<br>Independent Mobility Score<br>(Prezza et al., 2001)	**Correlates**<br>Social Environment<br>(1) Maternal perception of social danger<br>(2) Maternal perception of the positive potentiality of outdoor autonomy<br>(3) Neighborhood relations<br>(4) Sense of community<br>Physical environment<br>(5) Presence of public parks in the area around the home<br>(6) size of the context in which participants lived<br>**Measurements**<br>Parental reports<br>**Instruments**<br>(1) Social Danger Perception Scale (Prezza et al. 2005)<br>(2) Perception of the Positive Potentiality of Outdoor Autonomy for Children Scale (Prezza et al. 2005)<br>(3) Neighborhood Relations Scale (Prezza & Pacilli, 2002)<br>(4) Italian Sense of Community Scale (Prezza et al. 1999)<br>(5) two questionnaire items<br>(6) count of inhabitants	- Age of the child, perception of social danger (-0.28) and perception of positive potentiality of outdoor autonomy for children (0.36) are strongly related to CIM.<br>- Being female significantly increase maternal perception of danger

**2**	Broberg et al., 2013<br><br>Helsinki, Finland	**Study design**<br>Cross-sectional<br>**Duration**<br>Autumn 2009<br>**Sample**<br>n = 901<br>mean age n.r./11 and 14 yrs<br>gender n.r.<br>RR: n.r.	**Definition**<br>CIM destination<br>**Measurements**<br>Children's self-reports<br>**Instruments**<br>Internet-based softGIS survey (Kahila & Kyttä, 2009; Kyttä, 2011)<br>(1) Marking of meaningful places on a map<br>(2) Question for Independent mobility: 'With whom do you travel to this place?'	**Correlates**<br>Physical environment<br>(1) Principal Components (PC):<br>PC 1: densely built up residential areas (# population, # housing units/hectare, % land cover apartm. build.)<br>PC 2: Mainly single-family housing (# buildings, % land cover single fam. Housing, % land cover semi-detached)<br>PC 3: Traffic dominance (% land cover traffic areas, # intersections, land use mix, % land cover green)<br>PC 4: Remote places (Dist. to nearest recreation facility, Dist. to nearest bus stop)<br>PC 5: Big building and public transport hubs (floor area ratio, # bus stops)<br>(2) distance to home<br>**Measurements**<br>Objective Measures<br>**Instruments**<br>14 GIS-based measures within a 50-m buffer zone around each meaningful place	- Higher scores on PC 2 and PC 4 increase CIM significantly (OR=1.271, 95%CI: 1.173-1.378, p<0.001; OR=1.174, 95%CI: 1.088-1.266, p<0.001)<br>- PC 1 increase CIM (OR=1.156, 95%CI: 1.062-1.259, p=0.0009)<br>- the higher loading on PC 5 the smaller the probability of IM (OR=0.76, 95%CI: 0.677-0.855, p<0.001)<br>- PC 3 was not associated with CIM<br>- distance from home is significantly associated with IM for all urban structure components
**3**	Buliung et al., 2017<br><br>Toronto, Canada	**Study design**<br>Cross-sectional<br>**Duration**<br>n.r.<br>**Sample**<br>n = 1035<br>11 yrs/9-13 yrs<br>50% F<br>RR: n.r.	**Definition**<br>CIM destination<br>**Measurements**<br>Children's self-reports<br>Parental report<br>**Instruments**<br>(1) Activity-travel survey<br>(2) Mapped routes	**Correlates**<br>Physical environment<br>(1) intersections crossed, missing sidewalks, maximum traffic, land use mix, traffic-calming, street-trees, urban structure, home-school distance, vehicles per licensed driver<br>Social environment<br>(2) Perceptions of the environment (safe area to walk alone, worried	To school:<br>- Individual and household characteristics accounted for 19% the model of variance (R^2:0.19)<br>Environmental characteristics<br>- Distance to school was the only significant environmental factor (p<0.01)<br>Perceptions:<br>- child's perception of living in a safe area to walk alone significantly increase IM (p<0.01)

				about strangers, fear of strangers, traffic around school, busy streets to cross)<br>**Measurements**<br>Objective Measures<br>Parental reports<br>**Instruments**<br>(1) Mapped routes and GIS analysis<br>(2) Activity-travel survey	From School:<br>- higher traffic volume deter IM ($p<0.05$)<br>- IM increase for children who perceive that they live in an area safe to walk alone in ($p<0.05$)<br>- Children of parents who somewhat agreed that traffic was too heavy around the school in the afternoon were less likely to walk independently ($p<0.05$)
**4**	Chaudhury et al. 2017<br><br>Auckland, New Zealand	**Study design**<br>Cross-sectional<br>**Duration**<br>2011-2012<br>May-June 2012<br>October-November 2013<br>**Sample**<br>n = 240<br>mean age n.r./9-12 yrs<br>*56.6%* F<br>RR: n.r.	**Definition**<br>CIM destination<br>**Measurements**<br>Children's self-reports<br>**Instruments**<br>Travel diaries	**Correlates**<br>Social environment<br>(1) Parental license of freedom (Mobility licenses)<br>Physical environment<br>(2) POS attributable index (POSAI, quantity and quality of POS)<br>**Measurements**<br>Parental reports<br>Objective Measures<br>**Instruments**<br>(1) Computer Administered Telephone Interview (CATI)<br>(2) GIS data and NZ-POST (Badland et al., 2010)	- children with higher parental license had greater odds of travelling independently ($p<0.001$)<br>**-** no associations was found between POSAI score and IM
**5**	Christian et al., 2015<br><br>Perth, Australia	**Study design**<br>Longitudinal (including cross-sectional analyses; Data of T4)<br>**Duration**<br>Feb. 2011-March 2012<br>**Sample**<br>n = 181<br>10.7 yrs./8-15 yrs.<br>55% F	**Definition**<br>CIM license<br>**Measurements**<br>Parental reports<br>**Instruments**<br>Questionnaire-Item: child is allowed to cycle or walk to 4 destination alone<br>(school, friends/family house,	**Correlates**<br>Physical Environment<br>(1) Park: distance to closest park by size category, count of parks, count of school grounds, presence of playgrounds, attractiveness score<br>School: count of primary schools, presence of secondary school<br>Shops: presence of shops for daily living<br>Traffic exposure: vehicles/day<br>Social environment	- Increasing access to local school grounds was associated with reduced independent mobility to the park (OR=0.77; 95%CI: 0.62–0.96)<br>- increasing distance to the closest large sized park was associated with reduced independent mobility to the park and school (OR=0.86; 95%C: 0.77–0.95, OR=0.88; 95%C: 0.79–0.99)<br>- increasing distance to the closest small sized park was associated with reduced independent

		RR: n.r.	park/oval/sporting field, local shop)	(2) poor neighborhood maintenance, social incivilities, graffiti and vandalism, property crime, loitering teenagers in public spaces dangerous or drunk driving, violent crime, parent perception of unsafe environment, poor collective efficacy, parenting social norms<br>**Measurements**<br>Objective Measures<br>Parental Reports<br>**Instruments**<br>(1) GIS data within a 1600m-buffer around each participants home<br>(2) Scale for neighborhood problems (Foster et al., 2010) and Scale for neighborhood safety (Australian Council for Educational Research, 2009)	mobility to the park (OR=0.85; 95%CI: 0.76–0.96)<br>- parent perception of an unsafe neighborhood for children to move around independently significantly decreased the odds of being independently mobile to school (OR=0.25; 95%CI: 0.09–0.70) and overall (OR=0.21; 95%CI: 0.06–0.70)<br>- None of the perceived neighborhood problems variables was significantly associated with overall IM<br>- If parents perceived that parenting social norms were unsupportive of independent mobility, the odds of their child's independent mobility to the local park (OR=0.64; 95%CI: 0.42–0.97) and shop (OR=0.56; 95%CI: 0.34–0.91) significantly decreased
**6**	Christian et al., 2016<br><br>Perth, Australia	**Study design**<br>Longitudinal (including cross-sectional analyses; Data of T4)<br>**Duration**<br>n.r.<br>**Sample**<br>n = 181<br>10.7 yrs/8-15 yrs<br>55% F<br>RR: n.r.	**Definition**<br>CIM license<br>**Measurements**<br>Parental reports<br>**Instruments**<br>Questionnaire-Item: child is allowed to cycle or walk to 4 destination alone	**Correlates**<br>Physical environment<br>(1) dog ownership<br>**Measurements**<br>Parental reports<br>**Instruments**<br>(1) dog ownership status (yes/no)	- dog ownership was associated with an increased odds of being independently mobile overall (OR=2.43; 95%CI: 1.03-5.74, $p<0.05$) but not with independent mobility to specific local destinations.
**7**	Cordovil et al. 2015<br><br>Portugal	**Study design**<br>Cross-sectional<br>**Duration**<br>Spring-time 2011<br>Spring-time 2012<br>**Sample**	**Definition**<br>CIM license<br>CIM destination<br>**Measurements**<br>Parental Report<br>Children's self-reports	**Correlates**<br>Social environment<br>(1) Mobility licenses<br>(2) Car ownership<br>Physical environment<br>(3) Home-school distance	- greater percentage of rural children than of urban school children are allowed to: go to places other than school on their own (53% vs. 45%) ($\chi2(1)=6.157$, $p=0.014$), go out after dark (17% vs. 10%) ($\chi2(1)=9.672$, $p=0.003$) and

		n = 1099<br>*11.4 yrs*/8-15 yrs<br>*53.4% F*<br>RR: 65.4%	**Instruments**<br>(1) Mobility licenses<br>(2) Portuguese Version of International Child Independent Mobility Questionnaire	(4) School area (urban vs. rural)<br>**Measurements**<br>Objective Measures<br>Parental reports<br>**Instruments**<br>(1) several sources of demographic and socio-economic information available in the official web sites of the local council and/or parishes, Statistics Portugal website<br>(2) Parents Questionnaire - Mobility license Score<br>(3) & (4) Parents Questionnaire	cycle on main roads alone (43% vs 27%) ($\chi 2(1)=20.170$, $p<0.001$)<br>- More urban than rural school children are allowed to travel on local buses alone (40% vs 29%) ($\chi 2(1)=11.295$, $p=0.001$)<br>- The percentage of children allowed to cross main roads (urban: 62%, rural: 67%) and to come home from school alone (urban: 48%, rural: 48%) was similar for urban and rural children.<br>- Rural children engage in more independent activities during the weekend ($t(541) = 4.82$, $p < .001$).<br><br>Multiple logistic regression:<br>- Distance from home to school is a strong predictor for IM<br>- being older and living in a household with no access to a car increase IM<br>- the number of mobility licenses is also an important variable predicting IM<br>- in the regression rural/urban location was not a significant predictor of IM on the school journey and on weekends ($p=0.059$)
**8**	Fyhri et al., 2009<br><br>Norway	**Study design**<br>Cross-sectional<br>**Duration**<br>2005<br>**Sample**<br>n = 1775<br>*8.9 yrs*/6-12 yrs<br>*48% F*<br>RR: 62%	**Definition**<br>CIM destination<br>**Measurements**<br>Children's self-reports<br>**Instruments**<br>Mobility Index	**Correlates**<br>(1) Physical environment (distance to sport and school, place of residence, local traffic situation, proportion of way to school with footpath, parent's experience pf traffic safety on way to school, stranger danger, children's safety experience, parent's car use, number of cars in household)<br>**Measurements**<br>Children's self-reports<br>Parental reports<br>**Instruments**	Structural equation Model:<br>- most influential children's age (0.30) and distance to school (-0.25)<br>- Time of year (-0.08), distance to leisure (sport) activities<br>(-0.08), experience of traffic safety on the way to school (0.08), experience of other types of safety on road to school (0.08) and parents' car use frequency (-0.08) all make small (0.08) but significant contributions to the variation in independent mobility<br>- Parents' education level, place of residence (urban vs. rural), number of cars in the household and parents' number of working

				Norwegian national travel survey (NTS)	hours per week do not make significant contributions to explaining independent mobility<br>- local traffic situation (-0.14) and length of footpath to school (-0.23) both influence indirectly independent mobility through parents' perception of how safe the way to school is
**9**	Janssen et al., 2016<br><br>United States	**Study design**<br>Cross-sectional<br>**Duration**<br>November, 2014<br>**Sample**<br>n = 497<br>mean age n.r./ 6.9-11.9 yrs<br>50.7% F<br>RR: n.r.	**Definition**<br>CIM range<br>**Measurements**<br>Parental reports<br>**Instruments**<br>Online survey<br>Items for IM (Veitch et al., 2014)	**Correlates**<br>Social environment<br>(1) Neighborhood correlates (parent's perception of safety: traffic problems, traffic calming, safe for children, fear of crime)<br>(2) Parental physical activity (with child)<br>**Measurements**<br>Parental reports<br>**Instruments**<br>(1) Neighborhood Safety Score (Carver et al., 2008)<br>(2) The Activity Support Scale for Multiple Groups (Davison et al., 2011)	- the children's age, their parent's perception that their neighborhood is safe for children (OR=4.24, 95%CI: 2.68-6.70), and their parent's perceived fear of crime in their neighborhood (OR=0.41, 95%CI: 0.27-0.62) were the significant correlates of independent mobility ($p<0.05$)<br>- no significant association was found for gender, number of parents in household and traffic calming
**10**	Johansson, M., 2006<br><br>Lund & Malmö, Sweden	**Study design**<br>Cross-sectional<br>**Duration**<br>n.r.<br>**Sample**<br>n = 357<br>9.6 yrs/8-11 yrs<br>50% F<br>RR: 67%	**Definition**<br>CIM licenses<br>CIM range and travel mode<br>**Measurements**<br>Parental reports<br>**Instruments**<br>1-week travel diary	**Correlates**<br>Social environment<br>(1) Household characteristics (car access)<br>(2) Parents' attitudes towards travel modes<br>(3) Individual parental factors (environmental trust, interpersonal trust, need to protect)<br>(4) Sense of community<br>Physical environment<br>(5) Physical environment (quality of traffic environment, design of foot	- trust in environment and in road users was correlated with IM<br>- older age, maturity and less parental need to protect were positively associated with IM<br>- quality of traffic environment correlated with positively with IM<br>- quality of cycle and footpaths did not correlate with the attitude toward independent travel<br><br>Independent Journey

				and cycle paths, general maintenance of the neighborhood)<br>**Measurements**<br>Parental reports<br>Expert reports<br>**Instruments**<br>(1) Attitude Scale<br>(2) & (3) Aspects of Küller's HEI-Model (1991)<br>(4) Sense of Community Scale (Chavis et al., 1986)<br>(5) expert checklist (Trucker-Cross & Küller, 2004)	- Attitude towards IM, traffic environment, car access and child's age correlated significantly with independent journeys
**11**	Kytta, M. 2004<br><br>Finland<br>Belarus	**Study design**<br>Cross-sectional<br>**Duration**<br>1994-1999 (Finland)<br>1997 (Belarus)<br>during the last two weeks of May<br>**Sample**<br>n = 223<br>8.1 yrs/8-9 yrs<br>56% F<br>RR: 80% Finnish parents, 86% Belarusian parents, 93% Finnish children, 100% Belarusian children	**Definition**<br>CIM licenses<br>CIM destination<br>**Measurements**<br>Parental reports<br>Children's self-reports<br>**Instruments**<br>(1) Mobility License Questionnaire (Hillmann et al., 1990)<br>(2) Actual Mobility Scale	**Correlates**<br>Physical environment<br>(1) Communities (two countries: Finland and Belarus; urban, small town and rural environments, suburban and a town contaminated (Belarus))<br>Social environment<br>(2) Mobility License<br>**Measurements**<br>Objective Measures<br>Parental reports<br>**Instruments**<br>(1) degree of urbanization (four step scale)<br>(2) Mobility License Questionnaire (Hillmann et al., 1990)	Mobility licenses:<br>- The mobility license scale scores of the children living in different communities differed in Finland ($F(3, 65)=27.51$, $p<0.001$) and in Belarus ($F(5, 131)=22.7$, $p<0.001$)<br>- in both countries license scores were highest for children living in the least urbanized communities<br>- mobility licences were positively associated with actual mobility (0.31)<br><br>Actual Mobility:<br>- The communities in these countries differed also in the share of the actual mobility scale score (Finland, $F(3, 79)=8.4$, $p=0.001$; Belarus $F(5, 146)=12.2$, $p<0.001$).<br>- The actual mobility score of the children did not vary systematically according to the degree of urbanization of the communities.
**12**	Lam et al., 2014<br><br>Hong Kong, China	**Study design**<br>Cross-sectional<br>**Duration**<br>September-December 2002	**Definition**<br>n.r.<br>**Measurements**<br>n.r.<br>**Instruments**	**Correlates**<br>(1) Physical environment (car ownership, population density, median household income, school density, network density, road	- household car ownership ($t=-5.54$, $df=2108$, $p<0.01$) had a negative effect on CIM<br>- children living in sprawl areas had greater IM ($x^2=34.06$, $df=2$, $p<0.01$)<br>- Population ($t=-1.37$, $df=2108$, $p<0.05$)

		**Sample**<br>n = 2110<br>8.77 yrs/6-12 yrs<br>51.61% F<br>RR: n.r.	Travel characteristics Survey 2002 (TCS02)<br>- household-based information<br>- household-member-based information<br>- trip based information<br>- CIM: yes/no	safety: accidents, land use mix, distance to school)<br>**Measurements**<br>Objective Measures<br>**Instruments**<br>(1) Databases<br>Small Tertiary Planning Units (urban, sprawl and rural locales)<br>Social deprivation index (SDI)<br>Traffic Accident Database System (road accidents)	and network density (t=-2.27, df =2108, p<0.05) had a negative effect on CIM<br>- School density had a positive influence on IM (t=3.32, df=2108, p<0.05)<br>- road accidents involving children, the social deprivation level and land use mix were not significant<br>- Children living further away from schools were significantly less likely to have CIM (x^2=117.28, df=3, p<0.001)
**13**	Lin et al., 2017<br><br>Auckland, New Zealand	**Study design**<br>Cross-sectional<br>**Duration**<br>2011-2012<br>**Sample**<br>n = 233<br>9.8 yrs/8-13 yrs<br>57.1% F<br>RR: n.r.	**Definition**<br>CIM destination<br>**Measurements**<br>Parental reports<br>Children's self-reports<br>**Instruments**<br>(1) 75 item computer-aided telephone interview (Oliver et al., 2011)<br>(2) Children's 1-week travel diaries	**Correlates**<br>(1) Parents' neighborhood perception (perception of neighborhood safety, perception of neighborhood cohesion, perception of neighborhood connection, parental concerns of neighborhood environment)<br>(2) Physical environment (car availability, street connectivity, distance to school, destination accessibility)<br>**Measurements**<br>Parental reports<br>Objective Measures<br>**Instruments**<br>(1) 75 item computer-aided telephone interview (Oliver et al. 2011)<br>(2) Observational audits and GIS Neighborhood Destination Accessibility Index-Child (NDAI-C)	- Parents' perception of neighborhood cohesion (OR=0.26, 95%CI: .01-.52) and connection (OR=0.25, 95%CI: .04-.46) is associated significantly with IM (p<0.05)<br>- parent's perception of neighborhood safety was not associated with CIM<br>- Distance to school was associated significantly with IM (OR=-0.17, 95% CI: -.32 -<br>-.01, p<0.05)<br>- never/sometimes car availability (OR=0.70, 95%CI: .22-1.18) was significantly associated with more IM (p<0.05)

**14**	Lopes et al., 2014<br><br><br>Portugal	**Study design**<br>Cross-sectional<br>**Duration**<br>2011 (spring-time)<br>2012 (spring-time)<br>**Sample**<br>n = 321<br>9.8 yrs/range n.r.<br>*51% F*<br>RR: n.r.	**Definition**<br>CIM destination<br>CIM license<br>**Measurements**<br>Children's self-reports<br>Parental reports<br>**Instruments**<br>(1) Portuguese Version of International Child Independent Mobility Questionnaire<br>(2)Mobility licenses – Parents Questionnaire<br>(3) Actual mobility - Children's questionnaire	**Correlates**<br>(1) Urbanization degree<br>**Measurements**<br>Objective Measures<br>Parental reports<br>**Instruments**<br>(1) identification by Statistics Portugal web site	- mobility licenses are significantly associated with urbanization degree<br>- significant differences in children's independent travel from home to school between the three environments ($x^2(2)=18.703$, $p<0.001$)<br>- significant differences in children's independent travel home from school between the three environments ($x^2(2)=17.031$, $p<0.001$)<br>- Children from the moderately and non-urbanized environments took part in 3 independent activities whereas children from highly-urbanized environment took part in 2 ($F(2,549)=7.579$, $p=0.001$)
**15**	Mammen et al., 2012<br><br>Greater Toronto and Hamilton Area, Canada	**Study design**<br>Cross-sectional<br>**Duration**<br>n.r.<br>**Sample**<br>n = 1016<br>8.7 yrs/ 6-14 yrs<br>*51% F*<br>RR: 40.3%	**Definition**<br>CIM destination<br>**Measurements**<br>Parental reports<br>**Instruments**<br>(1) computer-aided telephone interview: travel mode (escorted vs. unescorted)	**Correlates**<br>(1) active school travel safety (route safety, traffic safety, stranger/bullies)<br>(2) distance to school<br>(3) car ownership<br>**Measurements**<br>Parental reports<br>**Instruments**<br>(1) 5point-Likert scale	- too many cars around school and strangers /bullies approaching child was negatively associated with IM<br>- short distance to school was associated with greater IM<br>- car ownership was negatively associated with IM
**16**	Mitra, et al., 2014<br><br>Toronto, Canada	**Study design**<br>Cross-sectional<br>**Duration**<br>April 2010- June 2011<br>**Sample**<br>n = 795<br>10.48 yrs/range n.r.<br>53% F<br>RR: 77.4%	**Definition**<br>CIM license<br>**Measurements**<br>Parental reports<br>**Instruments**<br>Take-home questionnaire survey	**Correlates**<br>(1) Parental attitudes toward transportation mode (Automobile lover vs. Active travelers)<br>(2) Perception of neighborhood environment (enough sidewalks, crosswalks and traffic lights, barriers/obstacles, drivers to fast, distance between intersections to short, lots of shops and restaurants, road are not attractive, stranger danger, safe neighborhood)	- child was less likely to have a higher level of CIM if the parent was worried about strangers (OR=0.49, 95%CI: 0.37-0.66, $p<0.001$)<br>- child was more likely to have a higher level of CIM when the parent perceived the residential neighborhood as safe (OR=1.77, 95%CI: 1.21-2.60, $p<0.01$).<br>- The child of a parent who preferred walking, cycling or transit as modes for travelling was more likely to have a higher CIM level (OR=1.28, 95%CI: 1.04-1.57, $p<0.05$).

				**Measurements**<br>Parental reports<br>**Instruments**<br>(1) & (2) Take-home questionnaire survey	Multivariate analysis:<br>- Age and sex most important factors associated with IM followed by adult perceptions of neighborhood environmental quality<br>- socio-economic characteristics of a household had a relatively smaller effect<br>- parental travel attitudes had the smallest contribution in explaining CIM
**17**	Prezza et al. 2001<br><br>Rome, Italy	**Study design**<br>Cross-sectional<br>**Duration**<br>n.r.<br>**Sample**<br>n = 251<br>9.41 yrs/7-12 yrs<br>47% F<br>RR: n.r.	**Definition**<br>CIM destination<br>**Measurements**<br>Parental reports (mother)<br>**Instruments**<br>Semi-structured interview<br>- autonomous urban mobility	**Correlates**<br>(1) Mother psycho-social characteristics (sense of community, neighborhood relationship, fear of crime, perception of neighborhood safety in general/traffic)<br>(2) Physical environment (condominium courtyards, building with entrances on private streets and homes adjacent to parks, new and old neighborhoods)<br>**Measurements**<br>Parental reports<br>**Instruments**<br>(1) semi-structured interview<br>- Sense of neighborhood safety (Santinello et al., 1998)<br>- Italian Sense of Community Scale (Buckner, 1988)<br>- Neighborhood Relation scale (Prezza et al., 1999)<br>(2) identification by researchers	- Children's demographic characteristics explained 27.5% of variance (age, birth order, gender; p<0.01)<br>- maternal psychosocial variables (traffic perception: ß=0.129, p<0.05, neighborhood relations: ß=0.196, p<0.001) another 4,6%<br>- physical environmental variables (courtyard: ß=0.379, p<0.001; living near a pear: ß=0.179, p<0.01; old/new neighborhood: ß=0.181, p<0.01) explained 11,7% of variance<br>- mother's sense of community, fear of crime and neighborhood safety did not influence IM
**18**	Santos et al.,<br>2013<br><br>Portugal	**Study design**<br>Cross-sectional<br>**Duration**<br>2010-2011<br>**Sample**	**Definition**<br>CIM destination<br>**Measurements**<br>Children's self-reports<br>**Instruments**	**Correlates**<br>(1) Parental Physical Activity<br>(2) Parental perception of neighborhood safety (sidewalk and	- parental total MET-minutes/week (ß=0.104, p=0.041) and the perception of sidewalk and street safety (ß=0.132, p=0.009) were significant predictors of children's independent

		n = 354<br>11.63 yrs/range n.r.<br>55.9% F<br>RR: 54%	11-Item Questionnaire for IM<br>(Page et al., 2009)	street safety, fear of stranger, crime and traffic safety)<br>**Measurements**<br>Parental reports<br>**Instruments**<br>(1) International Physical Activity Questionnaire (IPAQ, 2012)<br>(2) Neighborhood Environment Walkability Scale (Saelens et al., 2003)	mobility, accounting for 13.0% of the variance (p<0.001).<br>- fear of strangers, crime and traffic safety was not a significant contributor
**19**	Veitch, et al., 2017<br><br>Australia	**Study design**<br>Longitudinal<br>**Duration**<br>T1: 2010<br>T2: 2012<br>**Sample**<br>n = 184<br>12.0 yrs/range n.r.<br>55% F<br>RR: 45%	**Definition**<br>CIM destination<br>**Measurements**<br>Children's self-reports<br>**Instruments**<br>Child survey	**Correlates**<br>(1) Perceived neighborhood attributes (personal safety, road safety, satisfaction with the local neighborhood)<br>(2) Objective measures of neighborhood attributes (availability of parks, walking tracks and bike tracks, distance between home and school)<br>**Measurements**<br>Parental reports<br>Objective Measures<br>**Instruments**<br>(1) Mother's survey<br>(2) GIS data<br>- 800m pedestrian network buffer	Predictors of IM on the school journey<br>-proximity to walking tracks (OR=3.52, 95%CI: 1.25-9.95, p<0.05), neighborhood safety (OR=1.37, 95% CI: 1.11-1.69, p<0.01) and greater satisfaction with the local neighborhood (OR=1.26, 95% CI: 1.06-1.49, p<0.01) predict greater IM<br><br>Predictors of IM to local destinations<br>- satisfaction with the neighborhood is positively associated with IM (B=0.45, 95%CI: 0.04-0.86, p<0.05)<br>- concerns over road safety was negatively associated with IM (B=-0.34, 95%CI: -0.59- -0.09, p<0.01)
**20**	Wolfe et al., 2016<br><br>Oakland and Berkely, California, USA	**Study design**<br>Cross-sectional<br>**Duration**<br>2006-2007<br>**Sample**<br>n = 305<br>12 yrs/10-14 yrs<br>51% F<br>RR: n.r.	**Definition**<br>CIM license<br>**Measurements**<br>Parental Reports<br>**Instruments**<br>Independent Mobility Index	**Correlates**<br>(1) Social environment (child-centered social control, intergenerational closure, social cohesion and trust, parental perception of neighborhood crime and traffic safety)<br>(2) Physical environment (housing unit density, cash store density, liquor store density, grocery store	- Parent's perception of social cohesion (B=0.571, p<0.01) and parent's perception of safety from traffic in the neighborhood (B=0.328, p<0.01) is positively associated with higher IM<br>- Parental perception of child-centered social control, intergenerational closure and safety for crime had no association with CIM<br>- there was little evidence of association of the physical environment and CIM, only housing

		density, retail employment, intersection density)<br>**Measurements**<br>Parental Reports<br>Objective measures<br>**Instruments**<br>(1) Child-centered social control Scale, intergenerational closure Scale, social cohesion an trust Scale (Sampson et al., 1999), Neighborhood Environment Walkability Scale (Cerin et al., 2006)<br>(3) GIS data<br>- 400m, 800m and 1600m buffers around respondents' homes	unit density (B=0.361, p<0.05) was significantly positive related

Abbreviations: CIM = Children's independent mobility; GIS =geographic information systems; F = female; n.r. = no response; POS = Public open spaces; RR = response rate; vs = versus; yrs = years;

Value in *italics*: Value (e.g. mean age, or distribution of gender) was calculated

Characteristics of included studies on association between the environment and CIM with separated results for girls and boys (n=7)

No	Author<br>Year<br>Country	Study Design<br>Duration<br>Sample<br>(sample size, mean age/range, gender, response rate)	Dependent Variable (IM)<br><br>Definition<br>Measurements<br>Instruments	Independent Variable (Types of environmental Correlates)<br><br>Measurement<br>Instruments	Results II<br>Gender differences in correlates
1	Carver et al., 2014<br><br>Norfolk, UK	**Study design**<br>Cross-sectional and prospective<br>**Duration**<br>T1: April-July 2007<br>T2: April-July 2008<br>**Sample**<br>n = 1121<br>mean age n.r. /9-10 yrs<br>57% F<br>RR: 57%	**Definition**<br>CIM destination (school) and travel mode<br>**Measurements**<br>Children's self-reports<br>**Instruments**<br>Questionnaires	**Correlates**<br>(1) access to car<br>(2) Parental rules and social support<br>(3) Perceptions of neighborhood (sense of community, safe place, neighborhood walkability, traffic and safety concerns)<br>(4) Objective environmental measures (neighborhood characteristics: road density, proportion of 'A' roads, streetlight density, effective walkable area, connected node ratio area, junction density, land use mix, socioeconomic deprivation;<br>route to school characteristics: streetlight density, main roads, proportion within an urban area;<br>school characteristics: travel plan, walk to school characteristics, school's walking access, school's cycling access)<br>**Measurements**<br>Parents reports<br>Children's self-reports<br>Objective Measures<br>**Instruments**<br>(1) and (2) Questionnaire	Prospective association with change in IM between T1 und T2<br>- Parents often allowing their child to play outside anywhere within the neighborhood (AOR 3.14, 95%CI: 1.24–7.96) and household car access (AOR 0.27, 95%CI: 0.08–0.94) were associated longitudinally with boys walking/cycling independently to school.<br>- Land use mix (AOR 1.38, 95%CI: 1.06– 1.79), proportion of main roads in the neighborhood (AOR 0.67, 95%CI: 0.47–0.94) and parental encouragement for walking/cycling (AOR 0.40, 95%CI: 0.20–0.80) were associated longitudinally with girls walking/cycling independently to school

				(3) Sense of community score (Panter et al., 2010) and Neighborhood walkability score (Panter et al., 2010)<br>(4) GIS data	
2	Carver et al., 2012<br><br>Australia	**Study design**<br>Cross-sectional<br>**Duration**<br>October-November 2010 (spring)<br>**Sample**<br>n = 430<br>10.4 yrs/range n.r.<br>52% F<br>RR: 18.5%	**Definition**<br>CIM destination and travel mode<br>CIM licenses<br>**Measurements**<br>Children's self-reports<br>Parental reports<br>**Instruments**<br>(1) Independent Mobility Questionnaire<br>(2) Independent Mobility on Weekends Score<br>(3) Mobility licenses Score	**Correlates**<br>(1) Mobility License<br>(2) Rural/urban setting<br>**Measurements**<br>Parental reports<br>Objective Measures<br>**Instruments**<br>(1) Mobility licenses Score<br>(2) GIS data	- in urban areas IM of boys was higher than in rural areas (not significant)<br>- there were no differences between urban/rural location for independent activities on weekends<br>- there were no significant differences in mobility licenses according to urban/rural location<br>- each additional mobility license was associated with increased IM for boys (p<0.01)<br>- in urban areas mobility licenses were positively associated with IM for girls (p<0.001)
3	Carver et al.,<br>2013<br><br>Australia<br><br>England, UK	**Study design**<br>Cross-sectional<br>**Duration**<br>Duration:<br>August-October (Australia)<br>February- March (UK)<br>**Sample**<br>n = 784<br>10.4 yrs/range n.r.<br>50% F<br>RR: n.r.	**Definition**<br>CIM destination<br>**Measurements**<br>Children's self-reports<br>Parental report<br>**Instruments**<br>Questionnaire-Items	**Correlates**<br>(1) Mobility licenses<br>(2) Urban or rural location<br>**Measurements**<br>Parental reports<br>Objective Measures<br>**Instruments**<br>(1) Mobility License Score<br>(2) Categorization as Urban (Inner City, Urban or Suburban) and Rural (Small Town rural)	- number of mobility licenses granted was significantly associated with increased odds of IM for school and non-school journeys (p<0.001)<br>- No differences in mobility licenses for boys and girls<br>- no differences between independent school journeys in rural and urban areas in England<br>- differences in IM in rural and urban areas in Australia, significantly more in urban areas (p<0.05)
4	Foster et al., 2014	**Study design**<br>Cross-sectional	**Definition**<br>CIM destination	**Correlates**	- Parental fear of strangers was significantly (p<0.05) associated with less independent

	Perth, Australia	**Duration**<br>July-December 2007<br>**Sample**<br>n = 1231<br>*11 yrs*/10-12 yrs<br>*52% F*<br>RR: 69.4% school; 56.5% children; 89.6% parents	**Measurements**<br>Children's self-reports<br>Parental reports<br>**Instruments**<br>Independent Mobility Score (Villanueva, 2012)	(1) Social environment (neighborhood friendliness, other children in the neighborhood, Informal social control, fear of strangers, parental perception of traffic)<br>(2) Physical environment (neighborhood walkability)<br>**Measurements**<br>Parental reports<br>Children's self-reports<br>Objective Measures<br>**Instruments**<br>(1) Parent-report Items, Neighborhood Friendliness Scale (Villanueva et al. 2013)<br>(4) GIS data: school-specific walkability index and Questionnaire-Item (Parents)	mobility among boys and girls (boys OR=0.66 ;girls OR=0.65)<br>- parents of independently mobile children perceived more informal social control in their neighborhood (boys OR=1.31; girls OR=1.33).<br>- Neighborhood friendliness was associated with boy's and girl's IM ($p<0.001$)<br>- neighborhood walkability and parental perception of traffic had no significant influence on CIM for boys ($p>0.05$)<br>- for girls neighborhood walkability ($p<0.001$) and parents perception of traffic ($p=0.025$) is associated with IM
5	Ghekiere et al., 2017<br><br>Flanders, Belgium	**Study design**<br>Cross-sectional<br>**Duration**<br>November 2014 - January 2015<br>**Sample**<br>n = 1286<br>10.6 yrs/10-12 yrs<br>50.5% F<br>RR: 41 % schools; 52.3% parents	**Definition**<br>CIM range (cycling)<br>**Measurements**<br>Parental reports<br>**Instruments**<br>Online Questionnaire<br>- Distance child is allowed to cycle without adult supervision	**Correlates**<br>(1) Parents usual transportation cycling<br>(2) Urbanization level<br>(3) Perceived Neighborhood characteristics (land-use mix, public transit stop, recreation facilities, aesthetic qualities, traffic safety, crime safety, pedestrian safety, presence of cycling infrastructure, maintenance of cycling infrastructure, vegetation)<br>**Measurements**<br>Parental reports<br>**Instruments**<br>Online Questionnaire<br>(1) International Physical Activity Questionnaire (Craig et al., 2003)<br>(2) Questionnaire-Item	- Parental cycling was not associated with IM<br>- Urbanization level was not associated with IM<br>- no interactions were found for neighborhood characteristics and boys' IM<br>- if parents perceived their neighborhood environment as being safe from traffic, girls had higher IM levels (expB=1.17, 95%CI: 1.03-1.33, $p<0.05$)

				(3) Physical Activity Neighborhood Environment Survey (Sallis et al., 2010)	
6	Villanueva et al., 2012<br><br>Perth, Australia	**Study design**<br>Cross-sectional<br>**Duration**<br>July-December 2007<br>**Sample**<br>n = 1480<br>*11 yrs*/10-12 yrs<br>51.0% F<br>RR: 69.4% schools; 56.5 % children; 89.6 % parents	**Definition**<br>CIM destination<br>**Measurements**<br>Children's self-reports<br>Parental reports<br>**Instruments**<br>(1) Mapping Activity<br>(2) Independent Mobility Index (Villanueva et al., 2012)	**Correlates**<br>(1) Perceived environmental factors (neighborhood friendliness, parent perception of safe neighborhood crossing, parents lack of fear in their child's personal safety in their neighborhood)<br>(2) Objective environmental factors (Count of, and shortest distance to, visited destinations using active travel, count of available destinations within 800m of a child's home)<br>**Measurements**<br>Objective Measures<br>Parental reports<br>**Instruments**<br>(1) Questionnaire Items<br>(2) GIS data	- If parents reported that they lived on a busy road, the likelihood of children's IM decreased by 52% to 64% (boys p=0.039; girls p=0.023)<br>- if children (boys p=0.038; girls p=0.055) and their parents (boys p=0.047; girls p=0.000) were confident in the child's ability to actively travel without an adult, their likelihood of IM more than doubled<br><br>Boys:<br>- Independent mobility was 42% to 67% higher in boys with more retail shops (p=0.005) and recreation venues (p=0.029) within 800 m of their home and 75% to 82% lower in boys with more local community services (p=0.005) and shopping centers (p=0.024).<br><br>Girls:<br>- irrespective of the type of destination category, density of destinations was not associated with girls' IM<br>- girls' likelihood of IM more than doubled if they lived in a high walkable area (p=0.016) and if they perceive the local park as safe (p=0.015)
7	Villanueva et al., 2014<br><br>Perth, Australia	**Study design**<br>Cross-sectional<br>**Duration**<br>July-December 2007<br>**Sample**<br>n = 1480<br>*11 yrs*/10-12 yrs<br>52.3% F	**Definition**<br>CIM destination<br>**Measurements**<br>Children's self-reports<br>Parental reports<br>**Instruments**<br>Independent Mobility Index (Villanueva et al., 2012)	**Correlates**<br>(1) School-specific walkability (street connectivity, road volume exposure)<br>(2) Perceived parent environmental factors (home on a busy road, neighborhood friendliness, safety of neighborhood road crossing)	- child's and their parents' confidence in the child's ability to walk to the closest shop without adult supervision, the child's perception that it was safe to play at the park closest to their house and that there were lots of children their own age to hang out with locally increased the odds of being independently mobile<br>- Girl's but not boy's IM was positively associate with attending a school located in a walkable

	RR: school: 69.4%;<br>56.5 % children; 89.6<br>% parents		(3) Social cultural factors (fear of child's personal safety)<br>**Measurements**<br>Objective Measures<br>Parental reports<br>Children's self-reports<br>**Instruments**<br>(1) GIS data<br>(2) Questionnaire Items<br>(3) Questionnaire Items	environment (girls OR=1.96, p=0.002; boys OR=0.98, p=0.950)<br>- higher odds of IM was found among boys whose parents perceived neighborhoods roads to be safety (OR=1.37, 95%CI: 1.17-1.62, p=0.000)<br>- for girls: association of parental perception of living on a busy road and IM was mediated by parents' confidence in their child's ability to walk to closest shop without an adult
