# Supplementary material for: Social and physical environmental correlates of independent mobility in children: a systematic review taking sex/gender differences into account
Source: Int J Health Geogr. 2018 Jul 3;17:24. doi: 10.1186/s12942-018-0145-9 (PMC6029402; doi:10.1186/s12942-018-0145-9)
Supplement: Supplementary file 2 — Additional file 2. Quality Assessment for all studies included. The table shows the methodological quality rating of each criteria and the total quality score for all included studies. [file 12942_2018_145_MOESM2_ESM.pdf]

Additional file 2. Quality Assessment for all studies included

	**Introduction**	**Methods**								**Results**		**Study Quality**	
Study (authors, year)	Objectives	Study design	Study Population (sampling fame, distribution by age and gender, rural/urban)	Response Rate >80%	Non-responders	Appropriateness of Measurement	Standardized Methods_IM	Standardized Methods_Correlates	Statistics	Internally Consistent Results	Comprehensive Description of Results	**Overall study quality score (percentage)**	**Study quality rating**
Alparone et al., 2012	1	0.5	1	1	0	0.5	1	1	1	1	0.5	**8.5 (77.3)**	**High**
Buliung et al., 2017	1	0.5	1	0	0	1	0.5	1	1	1	0.5	**8.5 (77.3)**	**High**
Broberg et al., 2013	1	1	0.5	0	0	1	0.5	1	1	1	1	**8 (72.7)**	**High**
Carver et al., 2014	1	1	1	0	1	0.5	0	1	1	1	1	**8.5 (77.3)**	**High**
Carver et al., 2012	1	1	0.5	0	0	1	0	1	1	1	1	**7.5 (68.2)**	**Low**
Carver et al., 2013	1	1	0.5	0	0	1	0	1	1	1	1	**7.5 (68.2)**	**Low**
Chaudhury et al. 2017	1	0.5	1	0	0	1	1	1	1	1	0.5	**8 (72.7)**	**High**
Christian et al. 2015	1	0.5	0.5	0	0	1	1	1	1	1	1	**8 (72.7)**	**High**
Christian et al. 2016	1	0.5	0.5	0	0	1	1	1	1	1	1	**8.5 (77.3)**	**High**
Cordovil et al., 2015	1	0.5	0.5	0	0	0.5	1	1	1	1	1	**7.5 (68.2)**	**Low**
Foster et al., 2014	1	0.5	1	0	0	1	1	1	1	1	1	**8.5 (77.3)**	**High**
Fyhri et al., 2009	1	0.5	1	0	1	0.5	0	0	0.5	1	1	**6.5 (59.1)**	**Low**
Ghekiere et al., 2017	1	0.5	1	0	0	0.5	0.5	0.66	1	1	1	**7.16 (65.1)**	**Low**
Janssen et al., 2016	1	0.5	0.5	0	0	1	0.5	1	1	1	0.5	**7 (63.6)**	**Low**
Johansson, 2006	1	0.5	0.5	0	0	1	0	1	0.5	1	1	**6.5 (59.1)**	**Low**

	Introduction	Methods								Results		Study Quality	
Study (authors, year)	Objectives	Study design	Study Population (sampling fame, distribution by age and gender, rural/urban)	Response Rate >80%	Non-responders	Appropriateness of Measurement	Standardized Methods_IM	Standardized Methods_Correlates	Statistics	Internally Consistent Results	Comprehensive Description of Results	**Overall study quality score (percentage)**	**Study quality rating**
Kytta, 2004	1	1	0.5	1	0	1	1	1	1	0.5	1	**9 (81.8)**	**High**
Lam & Loo, 2014	1	0.5	1	0	0	0.5	0	1	1	1	1	**7 (63.6)**	**Low**
Lin et al., 2017	1	0.5	0.5	0	0	1	0	0.6	1	1	1	**6.6 (60.0)**	**Low**
Lopes et al., 2014	1	0.5	1	0	0	0.5	0	0	1	1	1	**6 (54.6)**	**Low**
Mammen et al., 2012	1	0.5	1	0	0	1	1	0	1	1	1	**7.5 (68.2)**	**Low**
Mitra et al., 2014	1	0.5	1	0	0	0.5	0	0.25	1	1	1	**6.25 (56.8)**	**Low**
Prezza et al., 2001	1	0.5	0.5	0	0	1	1	1	0	1	1	**7 (63.6)**	**Low**
Santos et al., 2013	1	0.5	0.5	0	1	1	1	1	1	1	1	**9 (81.8)**	**High**
Veitch et al., 2017	1	1	1	0	0	0.5	1	0.4	1	1	1	**7.9 (71.8)**	**High**
Villanueva et al., 2012	1	0.5	1	0	0	1	0.5	0.6	1	1	1	**7.6 (69.1)**	**Low**
Villanueva et al., 2014	1	0.5	1	0	0	0.5	0.5	1	1	1	1	**7.5 (68.2)**	**Low**
Wolfe & McDonald, 2016	1	0.5	0.5	0	0	1	1	0.83	1	1	1	**7.83 (71.2)**	**High**
